# A proposal for a CT driven classification of left colon acute diverticulitis

**DOI:** 10.1186/1749-7922-10-3

**Published:** 2015-02-19

**Authors:** Massimo Sartelli, Frederick A Moore, Luca Ansaloni, Salomone Di Saverio, Federico Coccolini, Ewen A Griffiths, Raul Coimbra, Ferdinando Agresta, Boris Sakakushev, Carlos A Ordoñez, Fikri M Abu-Zidan, Aleksandar Karamarkovic, Goran Augustin, David Costa Navarro, Jan Ulrych, Zaza Demetrashvili, Renato B Melo, Sanjay Marwah, Sanoop K Zachariah, Imtiaz Wani, Vishal G Shelat, Jae Il Kim, Michael McFarlane, Tadaja Pintar, Miran Rems, Miklosh Bala, Offir Ben-Ishay, Carlos Augusto Gomes, Mario Paulo Faro, Gerson Alves Pereira, Marco Catani, Gianluca Baiocchi, Roberto Bini, Gabriele Anania, Ionut Negoi, Zurabs Kecbaja, Abdelkarim H Omari, Yunfeng Cui, Jakub Kenig, Norio Sato, Andras Vereczkei, Matej Skrovina, Koray Das, Giovanni Bellanova, Isidoro Di Carlo, Helmut A Segovia Lohse, Victor Kong, Kenneth Y Kok, Damien Massalou, Dmitry Smirnov, Mahir Gachabayov, Georgios Gkiokas, Athanasios Marinis, Charalampos Spyropoulos, Ioannis Nikolopoulos, Konstantinos Bouliaris, Jaan Tepp, Varut Lohsiriwat, Elif Çolak, Arda Isik, Daniel Rios-Cruz, Rodolfo Soto, Ashraf Abbas, Cristian Tranà, Emanuele Caproli, Darija Soldatenkova, Francesco Corcione, Diego Piazza, Fausto Catena

**Affiliations:** Department of Surgery, Macerata Hospital, Macerata, Italy; Department of Surgery, University of Florida, Gainesville, FL USA; General Surgery I, Papa Giovanni XXIII Hospital, Bergamo, Italy; Trauma Surgery Unit, Maggiore Hospital, Bologna, Italy; Department of Surgery, Queen Elizabeth Hospital, Birminham, UK; Department of Surgery, UC San Diego Health System, San Diego, USA; Department of Surgery, Ospedale Civile, ULSS19 del Veneto, Adria, (RO) Italy; First Clinic of General Surgery, University Hospital St George, Plovdiv, Bulgaria; Department of Surgery, Fundación Valle del Lili, Hospital Universitario del Valle, Universidad del Valle, Cali, Colombia; Department of Surgery, Faculty of Medicine and Health Sciences, UAE University, Al-Ain, United Arab Emirates; Surgery 3 Unit, University Clinic for Emergency Surgery, Belgrade, Serbia; Department of Surgery, University Hospital Center, Zagreb, Croatia; General and Digestive Tract Surgery, Alicante University General Hospital, Alicante, Spain; 1st Surgical Department of First Faculty of Medicine, General University Hospital, Prague Charles University, Prague, Czech Republic; Department of General Surgery, Kipshidze Central University Hospital, Tbilisi, Georgia; Department of General Surgery, Centro Hospitalar São João, Faculdade de Medicina da Universidade do Porto, Porto, Portugal; Department of Surgery, Pt BDS Post-graduate Institute of Medical Sciences, Rohtak, India; Department of Surgery, Mosc Medical College, Kolenchery, Cochin India; Department of Surgery, Sheri-Kashmir Institute of Medical Sciences, Srinagar, India; Department of General Surgery, Tan Tock Seng Hospital, Tan Tock Seng, Singapore; Department of Surgery, Ilsan Paik Hospital, Inje University College of Medicine, Goyang, Republic of Korea; Department of Surgery, Radiology, Anaesthetics and Intensive Care University Hospital of the West Indies, Kingston, Jamaica; Department of Abdominal Surgery, Umc Ljubljana, Ljubljana, Slovenia; Surgical Department, General Hospital Jesenice, Jesenice, Slovenia; Department of General Surgery, Hadassah Medical Center, Jerusalem, Israel; Department of General Surgery, Rambam Health Care Campus, Haifa, Israel; Federal University of Juiz de Fora (UFJF) AND Faculdade de Ciências Médicas e da Saúde de Juiz de Fora (SUPREMA), Juiz de Fora, MG Brazil; Department of General Surgery, Trauma and Emergency Surgery Division, ABC Medical School, Santo André, SP Brazil; Emergency Surgery and trauma Unit, Department of Surgery, Ribeirão, Preto, Brazil; DEA, Umberto I University Hospital, Rome, Italy; Clinical and Experimental Sciences, Brescia Ospedali Civili, Brescia, Italy; General and Emergency Surgery SG Bosco Hospital, Turin, Italy; Department of Surgery, Arcispedale S. Anna, Medical University of Ferrara, Ferrara, Italy; Emergency Hospital of Bucharest, University of Medicine and Pharmacy Carol Davila Bucharest, Bucharest, Romania; General and Emergency Surgery Department, Riga East University Hospital “Gailezers”, Riga, Latvia; Department of General Surgery, King Abdalla University Hospital, Irbid, Jordan; Department of Surgery, Tianjin Nankai Hospital, Nankai Clinical School of Medicine, Tianjin Medical University, Tianjin, China; 3rd Department of Generał Surgery, Narutowicz Hospital, Krakow, Połand; Department of Primary Care & Emergency Medicine, Kyoto University Graduate School of Medicine, Kyoto, Japan; Department of Surgery, Medical School University of Pécs, Pécs, Hungary; Department of Surgery Hospital and Oncological Centre Novy Jicin, Novy Jicin, Czech Republic; Department of General Surgery, Numune Training and Research Hospital, Adana, Turkey; 2nd Surgical Unit, Santa Chiara Hospital, Trento, Italy; Department of Surgery, Hamad General Hospital, Doha, Qatar; II Cátedra de Clínica Quirúrgica, Hospital de Clínicas, Asuncion, Paraguay; Department of Surgery, Edendale Hospital, Pietermaritzburg, South Africa; Department of Surgery, Ripas Hospital, Bandar Seri Begawan, Brunei; Department of Surgery, University Hospital of Nice, University of Nice Sophia-Antipolis, Sophia-Antipolis, France; Department of Surgical Diseases, South Ural State Medical University, Chelyabinsk City, Russian Federation; Department of Surgery, Clinical Hospital of Emergency Medicine, Vladimir City, Russian Federation; 2nd Department of Surgery, Aretaieio University Hospital, Athens, Greece; First Department of Surgery, Tzanion General Hospital, Piraeus, Greece; 3rd Department of Surgery, Iaso General Hospital, Athens, Greece; Department of General Surgery, Lewisham & Greenwich NHS Trust, London, UK; Department of General Surgery, University Hospital of Larissa, Larissa, Greece; Department of General Surgery, North Estonia Medical Center, Tallinn, Estonia; Department of Surgery, Faculty of Medicine Siriraj Hospital Mahidol University, Bangkok, Thailand; Department of Surgery, Samsun Education and Research Hospital, Samsun, Turkey; Department of Surgery, Mengucek Gazi Training Research Hospital, Erzincan, Turkey; Department of Surgery, Hospital de Alta Especialidad de Veracruz, Veracruz, Mexico; Department of Emergency Surgery and Critical Care, Centro Medico Imbanaco, Cali, Colombia; Emergency Surgery, Faculty of Medicine, Mansoura University, Mansoura, Egypt; Department of Emergency Medicine and Surgery, Macerata Hospital, Macerata, Italy; Surgical Clinic, Ancona University Hospital, Ancona, Italy; Department of Laparoscopic and Robotic Surgery, Colli-Monaldi Hospital, Naples, Italy; Division of Surgery, Vittorio Emanuele Hospital, Catania, Italy; Emergency Department, Maggiore University Hospital, Parma, Italy

## Abstract

Computed tomography (CT) imaging is the most appropriate diagnostic tool to confirm suspected left colonic diverticulitis. However, the utility of CT imaging goes beyond accurate diagnosis of diverticulitis; the grade of severity on CT imaging may drive treatment planning of patients presenting with acute diverticulitis.

The appropriate management of left colon acute diverticulitis remains still debated because of the vast spectrum of clinical presentations and different approaches to treatment proposed. The authors present a new simple classification system based on both CT scan results driving decisions making management of acute diverticulitis that may be universally accepted for day to day practice.

## CT imaging in the diagnosis of acute diverticulitis

Diverticular disease is common in western countries, however the prevalence of colonic diverticulosis is increasing throughout the world, probably because of changes in lifestyle [1]. Diverticulitis is the most usual complications of diverticulosis, affecting 15-25% of patients [2]. Acute diverticulitis encompasses a variety of conditions, ranging from localized diverticular inflammation to fecal peritonitis. It has been usually divided in to uncomplicated and complicated basing on the extension of infection process to the peritoneum. Acute uncomplicated diverticulitis is now successfully treated in most patients by conservative management [3, 4].

In recent years the disease natural history and the individual physiology of local and/or systemic inflammatory response have been understood and minimally invasive strategies, have been applied to selected patients with more severe disease [5] despite the lack of well conducted randomized clinical trials [6]. As a consequence the choice of the surgical strategy has been often guided by the surgeon’s personal preference [7] rather than by available published medical literature. An accurate assessment of patients with acute diverticulitis by both clinical signs and radiological features is necessary to identify the best treatment for each patient before the treatment.

Over the last three decades, acute complicated diverticulitis has been divided traditionally into four stages according to the Hinchey classification. The Hinchey classification can only be accurately applied in patients who are operated on [8]. Therefore, there is a need to develop radiological staging systems to help in the management of all patients, especially as a significant proportion are managed without surgery or with radiological drainage.

However, in recent years the emergency management of acute sigmoid diverticulitis has evolved dramatically. Computed tomography imaging has become by now the gold standard in the diagnosis and staging of patients with acute diverticulitis. CT imaging with intravenous contrast has excellent sensitivity and specificity, reported as high as 98% and 99% [9, 10]. To improve image quality it should be performed with intravenous contrast medium injection (when acute renal failure is not present) and at least two phases (without contrast and portal venous phase). The utility of CT imaging goes beyond accurate diagnosis of diverticulitis; the grade of severity on CT may drive treatment planning of patients with acute diverticulitis [11–18]. CT is now the most useful tool to diagnose acute diverticulitis and associated to the clinical conditions and the physiological reserve of the patients it can objectively grade its severity into mild-moderate and severe diverticulitis [11].

Abdominal ultrasound is an alternative imaging modality that may be useful in patients with relative contraindications to CT scanning (pregnancy, renal insufficiency, and contrast allergy). CT is therefore superior to US, especially in the detection of free air and deeply located or small fluid collections and can drive the clinicians in the management plan [19]. Limitations of ultrasound include operator-dependency, poor assessment in obese patients and it may not be practical in patients with abdominal tenderness because the transducer probe requires compression. Some authors recommend a step-up approach with CT performed after an inconclusive or negative US [20, 21].

CT scan of the abdomen and pelvis is the most appropriate imaging modality in the assessment of suspected acute diverticulitis.

CT scan should be performed in all patients with suspected complicated acute diverticulitis. In these forms CT is superior to US, especially in the detection of free air and deeply located or small fluid collections.

### A proposal for a new classification

The appropriate management of left colon acute diverticulitis remains still debated because of the vast spectrum of clinical presentations and the different approaches to treatment proposed. Some trials are in progress and will further define the appropriate management of complicated diverticulitis [22].

Based on the surgical findings of abscesses and peritonitis, Hinchey et al. classically classified the severity of acute diverticulitis into four levels [8]. In order to correlate the classification with the therapeutic approach modifications of Hinchey original classification were developed [23, 24]. The authors present a new simple classification system based on both CT scan results that may drive clinicians in management of acute diverticulitis and that may be universally accepted for day to day practice. The new classification divides acute diverticulitis into 2 groups: uncomplicated and complicated.

In the event of an uncomplicated case of acute diverticulitis, the infection only involves the colon and does not extend to the peritoneum.

In the event of complicated IAI, the infectious process proceeds beyond the colon. Complicated acute diverticulitis is divided into 4 stages, based on the extension of the infectious process.

*Uncomplicated diverticulitis*

Diverticula, thickening of the wall, increased density of the pericolic fat

*Complicated diverticulitis*

**Figure 1:**
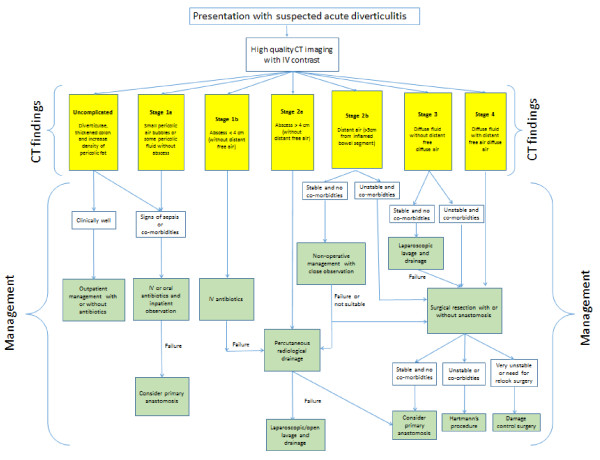
**Suggested management of acute diverticulitis in the emergency setting.**

1. A Pericolic air bubbles or little pericolic fluid without abscess
2. B Abscess ≤ 4 cm
3. A Abscess > 4 cm
4. B Distant air (>5 cm from inflamed bowel segment)
5. Diffuse fluid without distant free air (no hole in colon)
6. Diffuse fluid with distant free air (persistent hole in colon)The new classification is a starting point to stratify patients in well-defined stages. The definitive treatment for each stage can vary according to the clinical condition and functional reserves of the patient. The authors present the possible treatment strategies for each stage basing on both the clinical conditions of the patient and the presence of severe/multiple comorbidities (Figure 1).

### Uncomplicated acute diverticulitis

**Figure 2:**
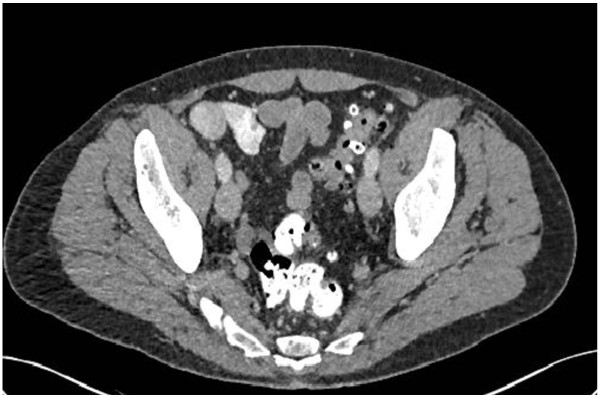
**Slightly thickened sigmoid diverticular disease, without abscess or perforation.**

Uncomplicated diverticulitis is a confined inflammatory process. CT findings include diverticula, thickening of the wall and, increased density of the pericolic fat (Figure 2). Patients with uncomplicated diverticulitis usually have an indolent course with a low incidence of subsequent complications. Complicated recurrence after an uncomplicated episode of diverticulitis is rare (<5%) and that age at onset younger than 50 years and 2 or more recurrences do not increase the risk of complications [25].

The efficacy of antibiotic use in acute uncomplicated diverticulitis, is a point of controversy in the medical community. Recent randomized studies found that antibiotic treatment was not superior to not antibiotic therapy in patients with mild unperforated diverticulitis, in terms of obtaining clinical resolution and preventing recurrence of diverticulitis [26–28]. The majority of uncomplicated diverticulitis can be safely managed on an outpatient basis unless they have risk factors (immunosuppression or multiple comorbidities) or signs of sepsis. In a retrospective analysis, Etzioni et al. [29] found that outpatient treatment was effective for the vast majority (94%) of patients suffering from acute diverticulitis. A recent systematic review on outpatient management of acute uncomplicated diverticulitis was recently published [30]. The authors concluded that current evidence suggests that a more progressive, ambulatory-based approach to the majority of cases of acute uncomplicated diverticulitis is justified. Patients unable to take oral fluids, who have significant comorbid conditions or presenting signs of sepsis should be treated as inpatients even if Rodríguez-Cerrillo et al. [31] have shown recently that also elderly people with co-morbidities can be safely treated at home without hospital admissions. The DIVER trial [32] recently stated that outpatient treatment is safe and effective in selected patients with uncomplicated acute diverticulitis and allows important costs saving to the health systems without negative influence on the quality of life of patients with uncomplicated diverticulitis.

*Patients with uncomplicated diverticulitis may be managed as outpatients. However patients unable to take oral fluids, who have significant comorbid conditions or presenting signs of sepsis should be treated as inpatients. Although most surgeons administer oral antibiotics in these patients, recent evidence suggests antibiotics do not confer any significant clinical benefit and can be avoided in patients without significant comorbid conditions or signs of sepsis. Patients should be clinical monitored as outpatients to assess for resolution of the inflammatory processes.*

### Stage 1 A

**Figure 3:**
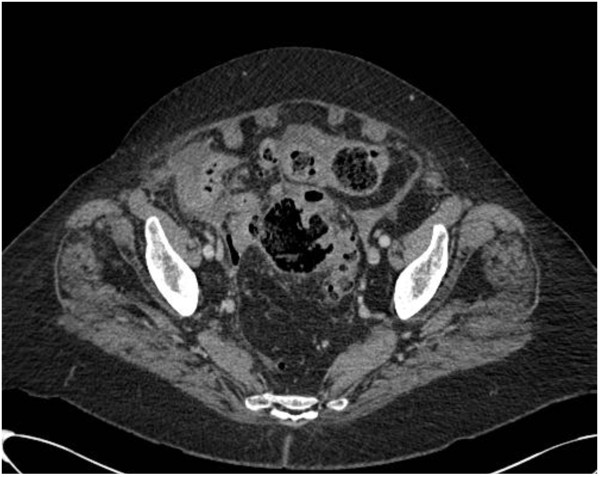
**Diverticular disease, colonic wall thickening, fat stranding and pericolic fluid and air bubbles.**

Stage 1 A diverticulitis is a confined inflammatory process that may include a microperforation but excludes an abscess, and/or peritonitis. CT findings include pericolic air in the form of air bubbles or little pericolic fluid without abscess. Pericolic air is defined as air bubbles or air collection within 5 cm of the inflamed bowel segment without distant air (Figure 3). Patients have left iliac fossa pain, initial localized tenderness and there are usually signs of sepsis. Small locules of pericolic air is a feature of a (micro) perforation of a diverticulum. Stage 1 A diverticulitis may progress to more complicated clinical forms if it is not treated promptly. Broad-spectrum antibiotics are therefore indicated in stage 1 A patients. Antibiotics should be always given to the patients with pericolic air or small fluid collection. Various antimicrobial regimens may be used in the treatment of acute diverticulitis in order to ensure complete coverage against Gram-positive, Gram-negative, and aerobic–anaerobic bacterial strains [33]. Antimicrobial regimens with beta-lactamase inhibiting antibiotics such as amoxicillin/clavulanic acid or ciprofloxacin plus metronidazole are appropriate for community acquired acute diverticulitis, although high rates of resistance to quinolones have reported in many countries. In critically ill patients or in immunocompromised patients broader-spectrum regimens should be used. An appropriate antimicrobial therapy given for an appropriate duration has minimal impact on the emergence of antimicrobial resistance.

Patients with CT findings of pericolic air and signs of sepsis and initial tenderness should require bowel rest, intravenous fluid hydration and empirical intravenous antimicrobial therapy. In these patients to optimize antimicrobial therapy and minimize hospital stay, antimicrobial therapy may be started initially intravenously and switched to oral therapy as soon as clinical conditions improve.

*Patients with pericolic air or small fluid collection should be managed by antimicrobial therapy. Patients with signs of sepsis and initial tenderness should demand bowel rest, intravenous fluid hydration and empirical intravenous antimicrobial therapy. In these patients intravenous antimicrobial therapy may be switched to oral therapy as soon as clinical conditions improve.*

*Clinical findings should be sufficient to monitor resolution of the acute episode. In patients who have persistent or recurrent clinical evidence of intra-abdominal infection after 4-6 days of therapy, new CT imaging should be undertaken.*

### Stage 1 B

Although the clinical presentation of acute left diverticulitis with associated abscess formation has increased in recent years, the therapeutic strategies for these patients are still debated. Deciding which patients with diverticular abscesses require percutaneous drainage rather than medical management therefore remains controversial [3]. Diverticular abscesses may be initially treated by intravenous antibiotics alone and/or percutaneous drainage, depending on the size of the abscess rather than the location (pelvic versus pericolic) [33–35]. The size of 4 cm (local severe diverticulitis) may be a reasonable limit between antimicrobial versus percutaneous drainage in management of diverticular abscesses. It is generally observed that abscesses with a size of up to 4 cm seem to respond better to intravenous antibiotics alone [33, 36, 37]. After initial antimicrobial therapy, no improvement of clinical conditions or rapid deterioration of clinical conditions suggest percutaneous drainage.

*Diverticular abscesses having a diameter of less than 4 cm may be treated by antibiotics alone. Broad-spectrum intravenous antibiotics and bowel rest are initially demanded. If antimicrobial treatment fails percutaneous drainage is suggested. CT scan should be repeated to demonstrate the resolution of the abscess. In patients who have persistent or recurrent clinical evidence of intra-abdominal infection after 4–6 days of therapy, CT imaging should be undertaken.*

Perforation with localised abscess collection is an uncommon presentation of colonic malignancy, and it may mimic complicated diverticular disease [38]. After the inflammation from a new onset of diverticulitis has resolved, traditionally patients have undergone colonoscopy to rule out colon cancer. However, the need for routine colonoscopy has recently been questioned [17].

A recent study by Sallinen et al. [39] provides additional insight into this debate. The study enrolled 633 patients with CT-diagnosed acute diverticulitis. Of these patients, 97 underwent emergency resection, whereas 536 were treated conservatively, 394 of whom underwent colonoscopy. The findings showed 17 cancers (2.7 %) in patients with an initial diagnosis of acute diverticulitis. As shown by CT, 16 cancer patients (94 %) had abscess, whereas one patient had pericolic extraluminal air but no abscess. Of the patients with abscess, 11.4 % had cancer mimicking acute diverticulitis. No cancer was found in the patients with uncomplicated diverticulitis.

*In patients with diverticular abscess treated conservatively, especially in those who do not respond to conservative management early colonoscopy (4–6 weeks) should be always planned*.

### Stage 2 A

**Figure 4:**
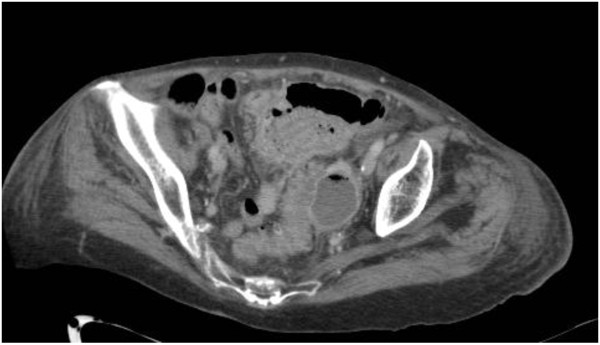
**Sigmoid diverticulitis with associated abscess formation.**

Acute left diverticulitis with abscesses > 4 cm respond better to percutaneous drainage with intravenous antibiotics (Figure 4). This is regardless of location (pelvic versus pericolic abscess). Percutaneous drainage has the advantage of allowing patients to avoid urgent operation [40–42]. The location of the abscess and its suitability and accessibility for radiologically percutaneous guided aspiration or drainage also needs to be carefully considered. In addition, patients with critical illness (for example severe sepsis or septic shock) may not be suitable candidates for radiological drainage and may be better treated with emergency surgery if they are fit enough. Antimicrobial therapy should always be given in addition to appropriate source control.

In selected cases when radiological drainage is not suitable or fails, laparoscopic/open peritoneal lavage and drainage, appears to be a useful option [25].

*Abscesses having a diameter of more than 4 cm are best treated by percutaneous drainage and intravenous antibiotics long as the patients do not have severe sepsis/septic shock. Whenever percutaneous drainage of the abscess is not feasible or not available, both surgical drainage of the abscess and surgical resection and anastomosis are viable options.*

*CT scan should be repeated to demonstrate the resolution of the abscess. In patients who have persistent or recurrent clinical evidence of intra-abdominal infection after 4–6 days of therapy, CT imaging should be undertaken.*

### Stage 2 B

**Figure 5:**
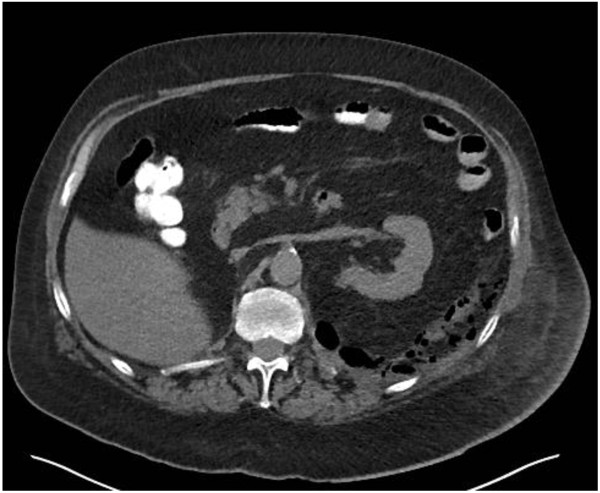
**Distant retroperitoneal free gas by perforated diverticular disease.**

A critical issue may be the CT presence of distant free air. Distant air is defined as air collections in the abdominal or retroperitoneal cavity with a distance >5 cm from the inflamed bowel segment (Figure 5).

Distant pneumoperitoneum is pathognomonic for sigmoid perforation even in absence of CT findings of diffuse peritoneal fluid.

Free air, seen on CT, has already been reported to be a useful predictor of failure of nonoperative treatment [10] even if Dharmarajan et al. [43] reported high success rate for nonoperative management in patients with diverticulitis and a pneumoperitoneum, excluding those with hemodynamic instability. In this study 136 patients were identified with perforated diverticulitis: 19 had localized free air, 45 had abscess <4 cm or distant free air measuring <2 cm, 66 had abscess >4 cm or distant free air >2 cm, and 6 had distant free air with free fluid. Thirty-eight patients (28%) required percutaneous abscess drainage and 37 (27%) required parenteral nutrition. Only 5 patients (3.7%) required urgent surgery at the time of admission, and 7 (5%) required urgent surgery for failed non-operative management.

An interesting retrospective cohort analysis was recently published to evaluate the safety and effectiveness of non-operative treatment of acute diverticulitis with extraluminal air [44].

A total of 132 patients underwent non-operative treatment and 48 patients were primarily operated on. Patients treated non-operatively were divided into 3 groups. Patients with pericolic air without abscess had a 99% success rate with 0% mortality. Patients with distant intraperitoneal air had a 62% success rate.

Whereas small series have demonstrated successful initial non-operative management of patients with acute complicated diverticulitis with perforation, even in the face of pneumoperitoneum, this strategy is reserved for highly selected stable patients without diffuse peritoneal findings with the goal of converting an emergent or urgent situation to one where an elective, single-stage operation can be performed.

*Patients with distant air (>5 cm from inflamed bowel segment) may be treated by conservative treatment in selected cases.*

*However it is associated with failure and may necessitate surgical operation. Careful clinical monitoring is mandatory. A CT scan should be repeated early on the basis of the clinical evolution.*

*Surgical resection and anastomosis with or without stoma is suggested in stable patients without multiple co-morbidities. Hartmann resection is suggested in unstable patients or in patients with multiple co-morbidities.*

### Stage 3

In recent decades, all cases of diffuse peritonitis have been treated by colonic resection.

Hartmann’s procedure has been the treatment of choice for decades, but in recent literature, a few interesting alternatives have emerged [45].

The restoration of intestinal continuity following Hartmann’s resection can be difficult and many patients cannot undergo the surgery due to medical co-morbidities; therefore, many of these patients remain with permanent stoma [46].

Primary colonic anastomosis, with or without defunctioning stoma, may be a safe alternative even in the presence of diffuse peritonitis, Several authors have consider a primary anastomosis an appropriate option in diffuse peritonitis, with or without defunctioning stoma with no difference in mortality or surgical site infection rate [47–56].

The first randomized trial of Hartmann’s procedure vs. primary anastomosis with ileostomy in patients with diffuse disease was published by Oberkofler et al. in 2012. It reported no difference in initial mortality, but a reduction in length of stay, lower costs, fewer serious complications and greater stoma reversal rates in the primary anastomosis group [56].

**Figure 6:**
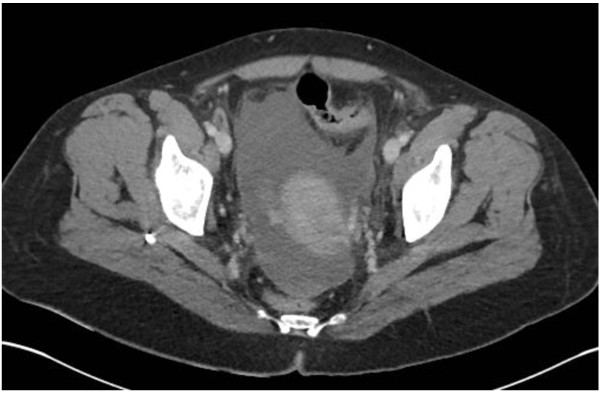
**Pelvic free fluid in patient with diffuse fluid and no distant air.**

We divided diffuse peritonitis in two stages. Stage 3 includes diffuse fluid without CT findings of perforation. In this stage CT does not reveal any evidence of distant free air (Figure 6). Fluid should be visualised in at least two distant abdominal quadrants.

A more conservative approach of laparoscopic peritoneal lavage and drainage has been described as an alternative to colonic resection by Myers et al. [57]. It can potentially avoid a stoma in the patients with diffuse peritonitis.

Pus is aspirated typically by laparoscopic access followed by abdominal lavage and the accurate placement of abdominal drains which remain for many days after the procedure [58].

Several series have been published, but evidence from a randomized controlled trial is still to be awaited [59].

In 2012 Karoui et al. [60] published a comparative study on postoperative outcomes of laparoscopic peritoneal lavage and open primary anastomosis with defunctioning stoma in the management of Hinchey 3 diverticulitis. In the management of Hinchey 3 diverticulitis, laparoscopic peritoneal lavage did not result in excess morbidity or mortality, it reduced the length of hospital stay and avoided a stoma in most patients.

Recently a retrospective analysis of 77 patients requiring emergency surgery for generalized peritonitis identified from a prospective database was published by Rossi et al. [61] to evaluate effectiveness of laparoscopic lavage. Laparoscopic assessment was considered in all of the hemodynamically stable patients, and laparoscopic lavage was performed according to intraoperative strict criteria. Forty-six patients who underwent laparoscopy presented a purulent generalized (Hinchey III) peritonitis and were examined under the intention-to-treat basis to perform a laparoscopic lavage. Thirty-two patients (70%) had no previous episodes of diverticulitis. Thirty-six patients (78.0%) had free air on a CT scan. The conversion rate was 4%. The feasibility of the method was 96.0%, and its applicability was 59.0%. Median operative time was 89 minutes (range, 40–200 minutes). Postoperative morbidity was 24.0%, and the mortality rate was 0%. Five patients failed to improve after this method of treatment and required re-operations. The effectiveness of the procedure was 85% (95% CI 76–93).

In 2013 a Dutch retrospective analysis of 38 patients treated by laparoscopic lavage was published. In 31 patients laparoscopic lavage controlled the sepsis. These patients had low mortality (1 died), acceptable morbidity and relatively rapid recovers. However, in the remaining 7 patients laparoscopic lavage did not control abdominal sepsis, two died of multiple organ failure and 5 required further surgical interventions (3 Hartmann resection, 1 diverting stoma and 1 perforation closure). One of these died from aspiration and the remaining four experienced prolonged complicated recoveries. The authors concluded that patient selection is of utmost importance and identification of an overt sigmoid perforation is of critical importance [62].

Additionally the authors noted that patients with diffuse peritonitis without perforation who had multiple co-morbidities, immunosuppression, a high C reactive protein level and/or a high Mannheim Peritonitis Index were at high risk of failure and concluded that a Hartmann procedure as a first step is the best option in these patients

Some trials are in progress and will further define the appropriate role of laparoscopic lavage and drainage [22].

Most recently first data of a randomized controlled multicenter trial to evaluate treatment for acute diverticulitis with purulent peritonitis (DILALA study) were published. Initial diagnostic laparoscopy showing Hinchey III was followed by randomization between laparoscopic lavage and colon resection and stoma. Clinical data was collected up to 12 weeks postoperatively. Eighty-three patients were randomized. 39 patients in laparoscopic lavage and 36 patients in the Hartmann procedure groups were available for analysis. Morbidity and mortality after laparoscopic lavage did not differ when compared with the Hartmann procedure. Laparoscopic lavage resulted in shorter operating time, shorter time in the recovery unit, and shorter hospital stay. The authors suggested that widespread implementation of the technique should await long-term results from the ongoing randomized trials [63].

*Laparoscopic peritoneal lavage is useful in managing clinically stable patients, without co-morbidities, who had generalized peritonitis without colonic perforation. It may avoid a stoma. No improvement or deterioration of the clinical condition following laparoscopic peritoneal lavage suggests the need for urgent colonic resection.*

*Surgical resection and anastomosis with or without stoma may be suggested for stable patients with no significant co-morbidities.*

*Hartmann resection should be carried out either in unstable patients (severe sepsis/septic shock) and/or in patients with multiple co-morbidities.*

### Stage 4

**Figure 7:**
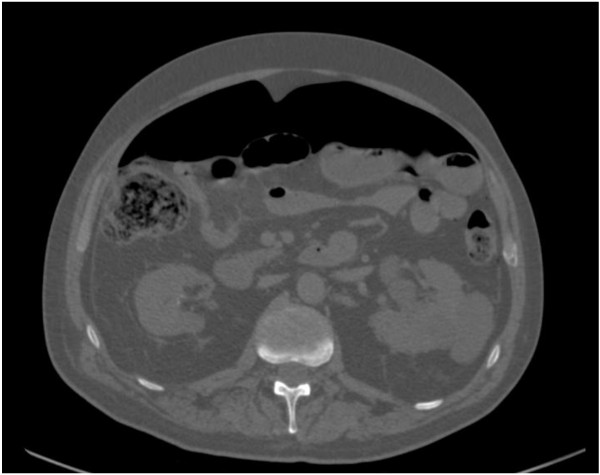
**Distant free air in patient with diverticulitis perforation.**

Diverticulitis perforation (Stage 4) (Figure 7) may be still treated by the classic Hartmann procedure even if several reports indicating that primary resection and anastomosis with or without diversion, have been reported as potential operative choices [64, 65]. Ultimately, this decision on the surgical strategy is left to the judgment of the surgeon, taking into account the clinical status of the patient including comorbidities, health of the remaining intestine, and extent of peritoneal contamination.

*Hartmann resection is still useful in managing diffuse peritonitis with signs of diverticular diffuse perforations, however in clinically stable patients with no co-morbidities primary resection with anastomosis with or without and diversion stoma may be performed.*

In specific cases of diverticulitis with perforation in unstable patients the ‘damage control surgery’ has become a valuable technique.

The term damage control surgery (DCS) for trauma patients was introduced in the 1990s. It was defined as initial control of haemorrhage and contamination, allowing for resuscitation to normal physiology in the intensive care unit and subsequent definitive re-exploration [66]. This concept can be equally applied to abdominal sepsis and the management of diverticular disease perforation in selected patients [67]. However, it should only be used in those rare instances, where the severe physiological compromise of the patient due to advanced generalised peritonitis preclude safe definitive management. Its overuse may potentially lead to increased morbidity due to the effects of multiple laparotomies and the sequalae of an open abdomen. A variety of temporary abdominal closure options have been described and include the classical Bogota bag, sandwich techniques, more modern and commercial Vacuum Assisted Closure (VAC) techniques. These techniques are advised in patients who require relook surgery or who are at risk of developing abdominal compartment syndrome [68].

The damage control surgery in generalized peritonitis can be performed in different ways [69–71]. In some sense, the Hartmann’s resection is a very good damage control procedure. In critically ill patients the operation can be staged. For example, the bowel can be resected and if the patient is too unwell for stoma formation, definitive intervention can be performed in 24–48 hours after appropriate resuscitation and stabilisation on the ICU. A retrospective study by Ordonez et al. [72] suggested that a deferred primary anastomosis (DPA) in patients with secondary peritonitis managed with staged laparotomies may allow eventual restoration of intestinal continuity during the same hospital stay. Among 112 patients there were 34 patients subjected to DPA and 78 to diversion. Fistulas/leakages developed in three patients (8.8%) with DPA and four patients (5.1%) with diversion (p = 0.359). ARDS was present in 6 patients (17.6%) with DPA and 24 patients (30.8%) with diversion (p = 0.149). There were 30 patients (88.2%) with DPA and 65 patients (83.3%) with diversion discharged alive (p = 0.51). There were not statistical significant differences between groups among survivors regarding hospital length of stay, ICU length of stay, and days on mechanical ventilation.

In a prospective study 51 patients with perforated diverticulitis (stage III/IV) [71] were initially managed with limited resection, lavage and temporary abdominal closure by a vacuum-assisted closure device followed by second, reconstructive operation 24–48 hours later. Hospital mortality rate was 9.8%; 35 (76%) of patients were discharged with reconstructed colon, and 93% of patients live without a stoma at follow-up. By damage control concept, an acceptable hospital mortality rate and a high rate of bowel reconstruction at second look were achieved in patients with perforated diverticulitis and generalized peritonitis.

*Damage control surgery may be a useful strategy in clinically unstable patients with perforated diverticulitis (severe sepsis/septic shock).*

## Conclusions

Acute diverticulitis should be managed according to its severity. Management options include conservative management with antibiotic treatment, abscess drainage, laparoscopy, and open surgery. Although the management strategy depends on more factors such as peritonitis diffusion, clinical conditions and physiological reserve of the patient, this new simple classification system based on CT scan results, may drive decisions making in non operative and operative management of acute diverticulitis. and can help making critical decisions in patients having acute diverticulitis. A prospective study should be designed to validate it.
